# Ab Initio Molecular Dynamics Simulations of Aqueous LiTFSI Solutions—Structure, Hydrogen Bonding, and IR Spectra

**DOI:** 10.1021/acs.jpcb.3c06633

**Published:** 2024-01-18

**Authors:** Piotr Wróbel, Piotr Kubisiak, Andrzej Eilmes

**Affiliations:** Faculty of Chemistry, Jagiellonian University, Gronostajowa 2, 30-387 Kraków, Poland

## Abstract

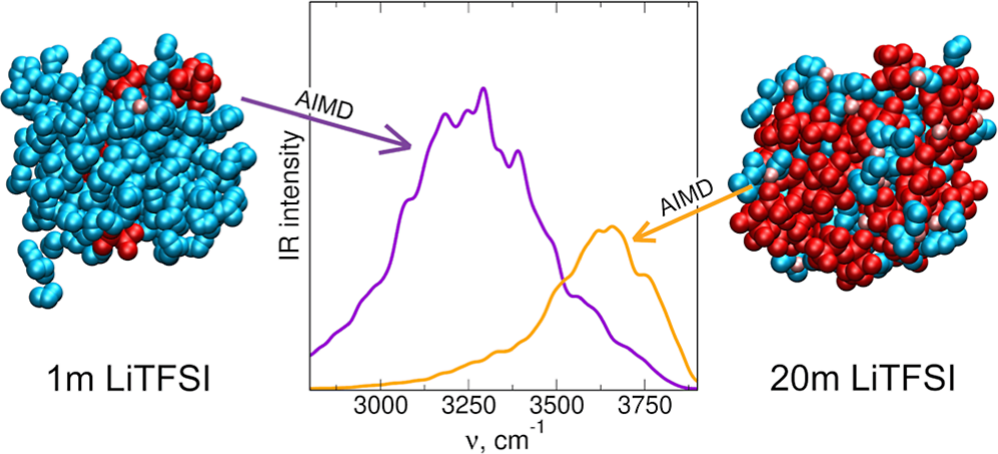

Simulations of Density Functional Theory-based ab initio molecular dynamics (AIMD) have been performed for a series of aqueous lithium bis(trifluoromethylsulfonyl)imide (LiTFSI) solutions with concentrations ranging from salt-in-water to water-in-salt systems. Analysis of the structure of electrolytes has revealed a preference of Li^+^ cations to interact with water molecules. In concentrated LiTFSI solutions, water molecules form small associates. The total number of hydrogen bonds (HBs) in the system decreases with salt concentrations, with bonds to water acceptors being only partially replaced by interactions with TFSI anions. Infrared (IR) spectra in the region of the O–H stretching frequency calculated from AIMD trajectories are in good agreement with experimental data. Statistics of oscillations of individual O–H bonds have shown correlations between vibrational frequencies and the structure of HBs formed by water. The changes in the IR spectrum have been related to the varying contributions of different local environments of the water molecules. The abundances of the three spectral components calculated from the simulations agree well with the decomposition of the experimental IR spectra reported in the literature.

## 1 Introduction

Energy storage technologies play a critical role in addressing the challenges of rising energy demand in a sustainable economy. Since their commercial introduction, lithium-ion batteries have emerged as very successful devices, with applications ranging from portable electronics to electric vehicles.^1−3^ However, these batteries commonly employ electrolytes based on organic liquids, which pose safety risks because of their flammability. Moreover, the use of organic solvents raises environmental concerns related to battery processing and recycling. These environmental and safety risks would be greatly reduced by aqueous electrolytes, but the narrow electrochemical stability window of water limits their commercial applications.

Recently, a new class of promising aqueous electrolytes was presented. It was demonstrated that in highly concentrated salt solutions in water, called water-in-salt (WiS) electrolytes, the electrochemical stability window is expanded, paving the way to stable and reversible aqueous electrolytes.^4,5^ A typical representative of WiS systems is LiTFSI salt dissolved in water, for which concentrations up to molality 21 m can be achieved.

Experimental investigations on the structure of LiTFSI WiS electrolytes involve small-angle X-ray scattering,^6^ small-angle neutron scattering,^6,7^ and spectroscopic techniques, in particular infrared (IR) or Raman spectroscopy.^6,8−12^ Experiments were supplemented and supported by classical molecular dynamics (MD) simulations, providing insights into the structure of the system and its relation to transport-related properties (viscosity, diffusion coefficients, conductivity, ion transport mechanism).^4,6−8,11−17^ Early works postulated nanoscale separation of water and salt domains in WiS and the presence of nanometric water channels.^7,8^ Later studies suggested that at the highest salt concentrations, ions form a network interpenetrating the whole system,^16^ whereas water molecules are rather isolated or associate into small aggregates.^11,12^ The latter conclusion was corroborated by ab initio molecular dynamics (AIMD) results for a dihydrate melt of two lithium salts.^18^ An interplay between ion–ion or ion–water interactions and hydrogen bonding is essential for the structure of the electrolyte and its IR spectrum.^11−13^

Most MD studies on concentrated salt solutions employ force field-based classical dynamics, allowing for a computationally efficient treatment of relatively large systems and calculations of their properties related to dynamics (correlation functions, diffusion coefficients, conductivity). On the other hand, the classical MD simulations are rather unsuitable for the reproduction of vibrational spectra. Works on WiS using first-principles AIMD are much less frequent; they comprise research on dihydrate melts^18^ or electrolytes based on Li salts in organic solvents.^19,20^ Just recently, a computational study has been published, reporting AIMD investigations of aqueous LiTFSI solutions.^21^ In addition to the structural analysis, AIMD simulations were used to calculate the IR and power spectra of two studied systems, one with low salt concentration, corresponding to a typical salt-in-water (SiW) solution and the other superconcentrated WiS electrolyte.^21^

Here, we present the results of another AIMD study on a series of LiTFSI/water systems with concentrations ranging from SiW to WiS electrolytes, aimed at the investigation of structural changes observed with changing molality of the solution. We want to extend the previous work of ref (21) by a detailed analysis of hydrogen bond (HB) networks in a series of solutions and its relation to the changes recorded in the IR spectrum in the region of O–H stretching vibrations. For the latter purpose, we used the method of correlating local environments of water molecules to the frequencies of O–H stretching oscillations, applied recently to ionic liquid/water solutions.^22^ This approach allows us to analyze both the structure of the electrolyte and the IR spectrum at the same level of computational methodology, facilitating comparison to the experimental data.

## 2 Computational Details

In this work, four LiTFSI/H_2_O solutions were studied, representing molal concentrations of the salt equal to 1, 5, 10, and 20 m. The number of atoms in the system varied from 718 to 741. Detailed compositions are given in Table 1. Packmol software^23^ was used to prepare the initial structures. For each concentration, two independent replicas of the system were simulated. Our strategy of combining classical and first-principles MD is to use the long run of the force field-based MD in order to obtain the equilibrated structure of the system, which is then improved during the AIMD simulations. To assess the quality of the reproduction of the structural properties, we compared the calculated static structure factor to experimental data. The computations of the vibrational spectrum require ab initio methodology; accordingly, the IR spectra were calculated from the AIMD trajectories.

**Table 1:** Compositions and Densities of the Studied Systems

molality (m)	no. of H_2_O molecules	no. of LiTFSI ion pairs	density (g/cm^3^)
1	222	4	1.133
5	167	15	1.413
10	122	22	1.587
20	83	30	1.719

Initial equilibration was performed using the classical MD and the NAMD v. 2.12 package.^24^ The force field parametrization for water molecules and TFSI anions was the same as in ref (22). van der Waals parameters for Li^+^ cations were assigned based on a parameter set for alkali metal ions in aqueous solutions.^25^ Force field parameters are listed in the Supporting Information. Classical MD simulations were performed for 200 ns in the NVT ensemble at 298 K; the equations of motion were integrated with a 1 fs time step. The particle mesh Ewald algorithm was employed to handle electrostatic interactions.^26^ The size of the periodic simulation box was set to reproduce the experimental density of the system, calculated from the molal and molar concentrations given in ref (9); resulting densities are listed in Table 1.

Next, structures from the classical MD trajectories were used as starting points for the Density Functional Theory (DFT)-based AIMD conducted in the CP2K program,^27,28^ employing the Perdew–Burke–Ernzerhof (PBE) functional with empirical dispersion correction D3,^29^ Goedecker’s pseudopotentials,^30^ and a molecularly optimized DZVP-MOLOPT-GTH basis set.^31^ A sample CP2K input file is included in the Supporting Information. AIMD simulations continued for 40 ps in the NVT ensemble at *T* = 298 K with a time step of 1 fs using the Nosé–Hoover thermostat. The last 30 ps (unless indicated otherwise) of the trajectories were used for analysis, and the results were averaged over both replicas. Plots of distribution functions were generated using TRAVIS software.^32^ The IR spectra were obtained from the recorded AIMD trajectories as Fourier transforms (FTs) of the dipole moment autocorrelation function. In the analysis of the effect of HBs on the O–H stretching frequency, FTs of the O–H distances for all water molecules were calculated, yielding the power spectra for the O–H oscillations. To produce smooth plots of the spectra, the individual peaks were convoluted with Gaussian curves with σ = 15 cm^–1^.

## 3 Results and Discussion

### 3.1 Structure of Electrolytes

**Figure 1:**
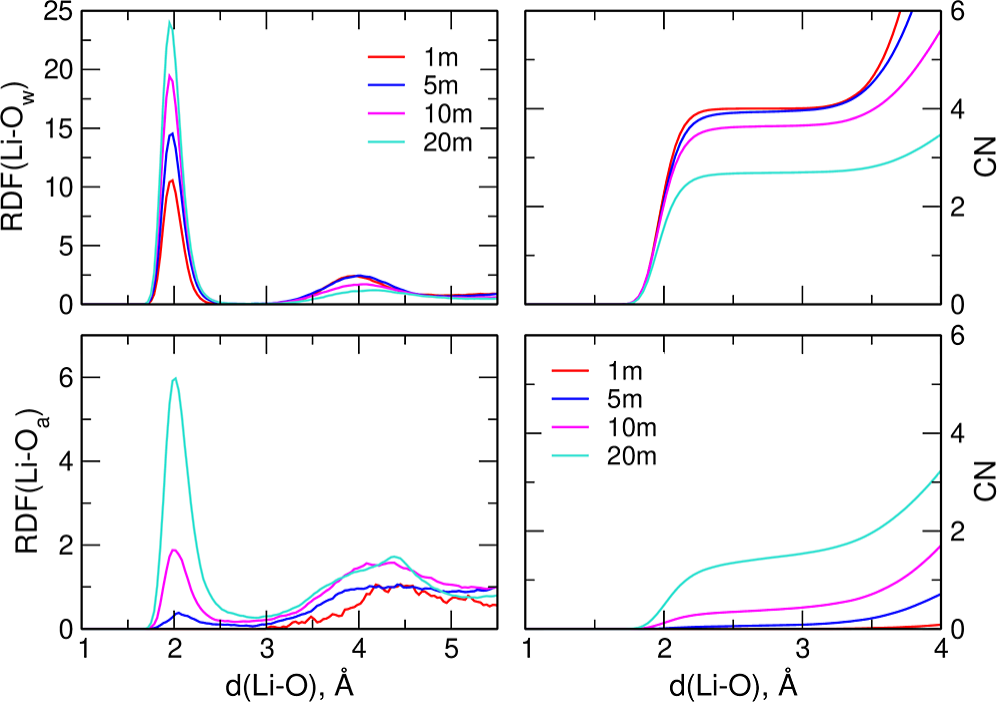
Radial distribution functions and integrated RDFs for Li–O_w_ and Li–O_a_ atom pairs.

We begin with the analysis of the structure of electrolytes with Li–water and Li–anion interactions. In Figure 1, we show the radial distribution functions (RDFs) and integrated RDFs (running coordination numbers, CNs) for Li–O_w_ and Li–O_a_ pairs, where O_w_ and O_a_ denote the oxygen atom from the water molecule and TFSI anion, respectively. The main maximum, corresponding to Li–O coordination, appears at 1.95–1.97 Å for Li–O_w_ or at 2.00–2.03 Å for Li–O_a_ atom pairs. The heights of the maxima increase with LiTFSI molality, but changes are much larger for the Li–O_a_ RDF. In this case, no maximum at 2 Å is visible for the 1 m solution, indicating that there are no cation–anion interactions at low salt concentrations and Li^+^ cations are solvated by water molecules. This observation is confirmed by the integrated RDFs, yielding CNs of the cations. In the 1 and 5 m solutions, there are, on average, 4.0 and 3.93, respectively, O_w_ atoms within the 2.75 Å from the Li^+^ ion. These values decrease to 3.64 and 2.69 in the 10 and 20 m electrolytes. At the lowest 1 m concentration, there are no O_a_ atoms at this distance, and their average number increases to 0.07, 0.39, and 1.45 in the 5, 10, and 20 m systems, respectively. It can be noted that the total number of O atoms coordinating the cation is almost concentration-independent: CN equals 4.00 at 1 and 5 m and increases slightly to 4.03 and 4.14 in the 10 and 20 m electrolytes, respectively; therefore, the total CN of the cation is barely affected by the salt concentration. A similar conclusion was drawn from the classical MD investigations of ion clusters in aqueous LiTFSI solutions.^16^

**Figure 2:**
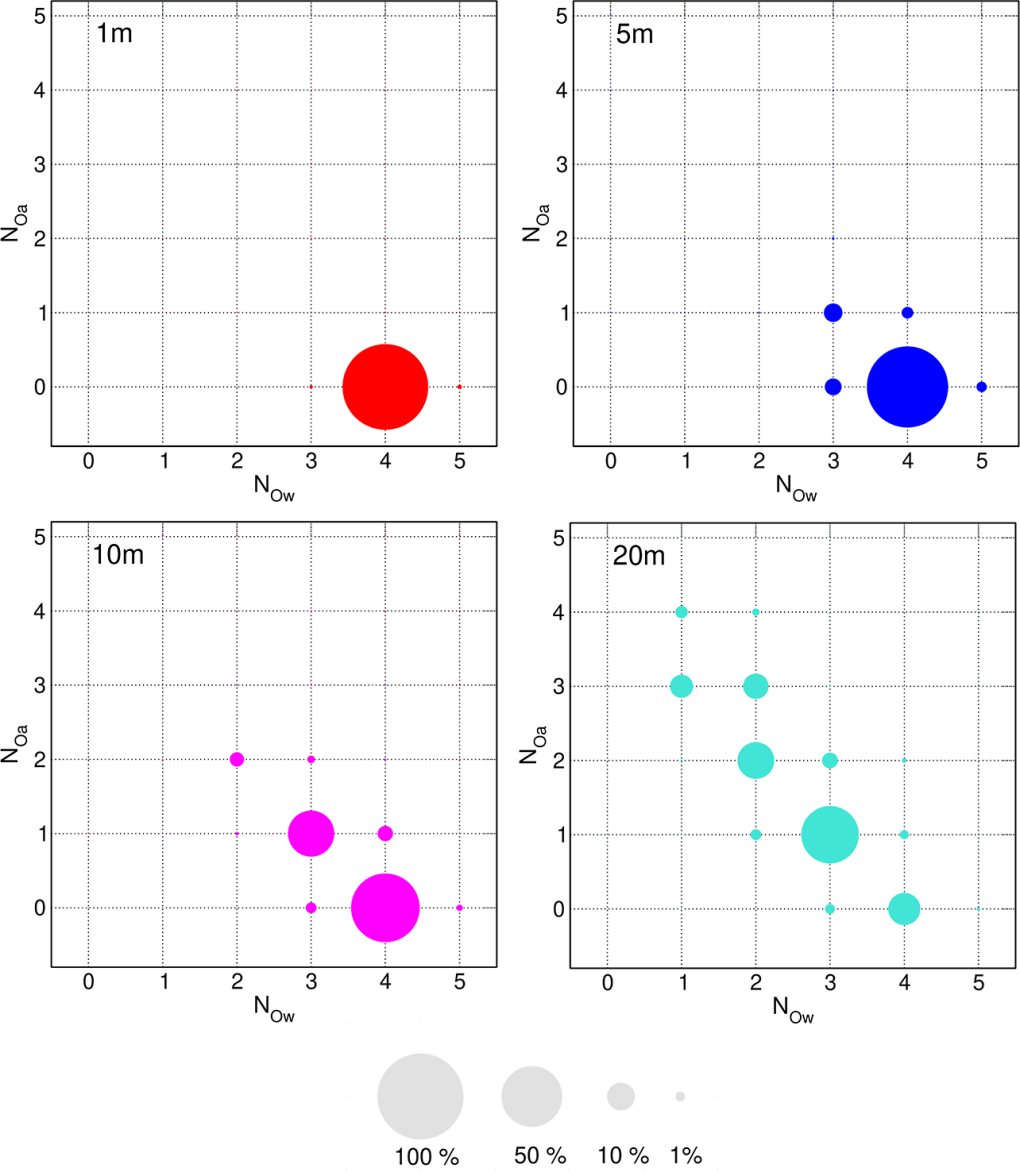
Probability of different Li^+^ coordinations to O_w_ and O_a_ atoms. The area of the circle is proportional to the abundance.

To gain more insights into the structure of the Li^+^ coordination shell, we calculated the abundance of different solvation environments, that is, the probability of combinations of average numbers of O_w_ and O_a_ atoms within a 2.75 Å distance from the cation. The results are presented in Figure 2. In the most dilute system (1 m), almost 100% of Li^+^ ions are tetrahedrally coordinated by four water molecules, and only about 0.2% are coordinated by three or five O_w_ atoms. In the 5 m electrolyte, interactions with four water oxygens are still dominating (90%), but about 6% of Li^+^ ions have at least one TFSI oxygen in the coordination shell, with most of them interacting with three O_w_ and one O_a_ atoms. The 4-fold coordination is the most preferred also in the 10 and 20 m solutions. In the former, 64% of Li^+^ interact solely with four O_w_ atoms, 29% with three O_w_ and one O_a_ atom, and 3% with two O_w_ and two O_a_ atoms. In the latter electrolyte, only 14% of the cations are coordinated to four water molecules; the most probable (45%) are the interactions with three O_w_ and one O_a_ oxygen. About 18% of Li^+^ in this system interacts with two O_w_ and two O_a_ atoms, 8% of cations are coordinated solely to three anion oxygens, and 7% to three O_a_ and one O_w_ atoms. From these data, we can conclude that in the SiW LiTFSI solutions, the most probable is the coordination of Li^+^ to four water molecules with no interaction with TFSI anions. This preference is still noticeable at a 10 m concentration. Only in the WiS systems do most Li^+^ cations have at least one anionic oxygen atom in the coordination shell. These findings are in agreement with classical MD and AIMD simulations, showing that the Li^+^ coordination with water oxygen atoms is preferred over the interactions with TFSI anions.^11,13,16,21^

**Figure 3:**
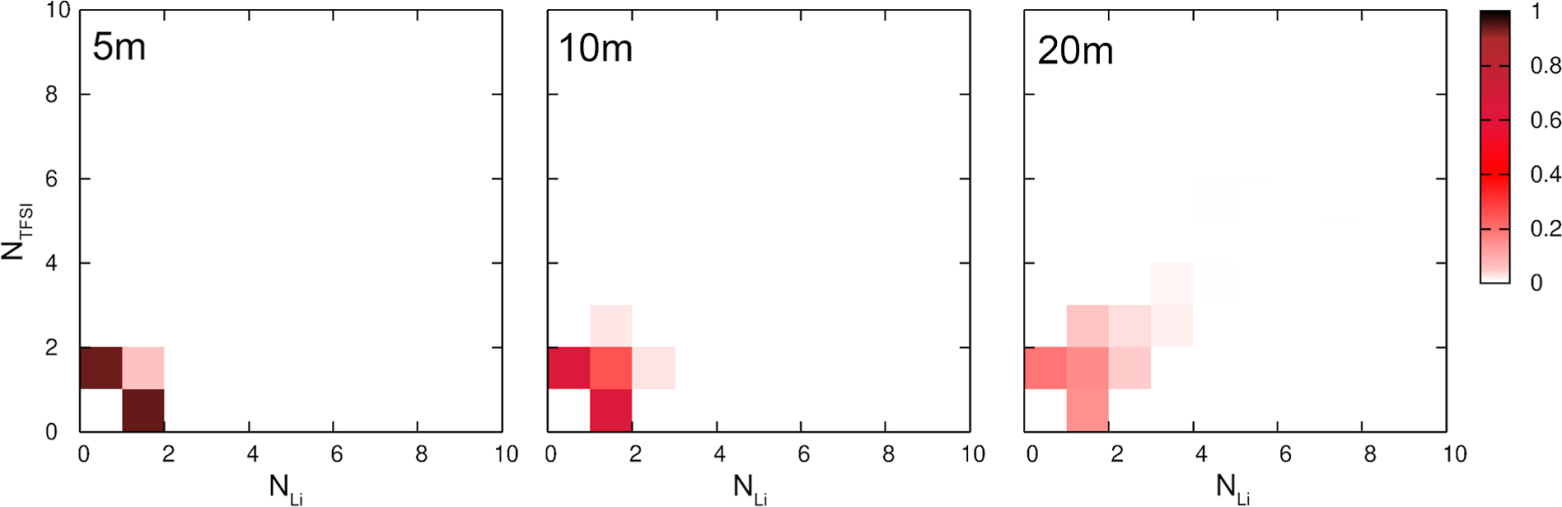
Abundance of ion aggregates in Li-TFSI electrolytes.

Another question regarding Li–TFSI interactions is the size of anion–cation associations formed in the electrolyte. To address this issue, we analyzed the last 15 ps of each trajectory, counting the number of anions and cations in the aggregates. The ion X belonged to the aggregate if its distance (defined as the distance between Li^+^ and the closest O atom of the TFSI anion) to a counterion in the aggregate was smaller than 2.75 Å. In the 1 m solution, there is no Li–O_a_ coordination, as shown by the CNs, and ions are isolated. Results for the three other concentrations are presented in Figure 3. About 94% of ions in the 5 m electrolyte are free, and the remaining 6% form neutral LiTFSI ion pairs. The percentage of free ions decreases to 65% at a 10 m concentration, with the amount of ion pairs increasing to 25%; the other ions exist in [Li(TFSI)_2_]^−^ and Li_2_TFSI^+^ triplets. Finally, in the WiS 20 m electrolyte, abundances of free ions and ion pairs are similar (15–19%), about 10% of ions are involved in ion triplets, and the remaining ions form larger aggregates, most of them with charge between −1 *e* and 1 *e*. The size of these aggregates reaches 20 ions, but their abundance decreases rapidly with the number of ions.

**Figure 4:**
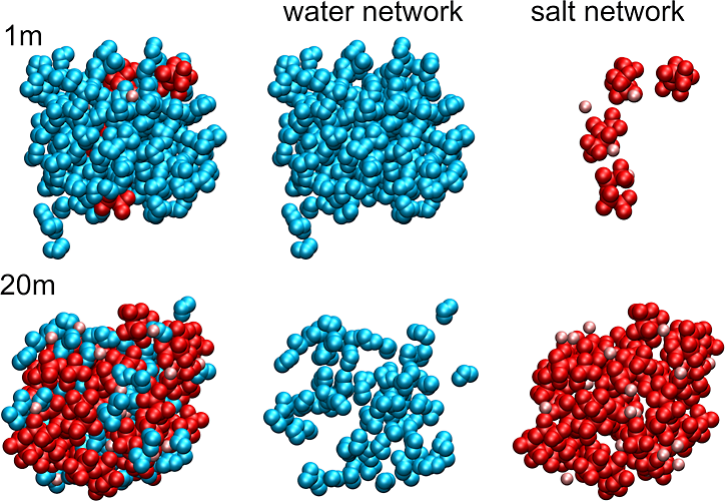
Snapshots of selected frames from the AIMD trajectories for 1 and 20 m LiTFSI solutions with a decomposition into water and salt network. Water molecules—blue, TFSI anions—red, and Li cations—pink.

The limited size of the system and short simulation time in AIMD make it difficult to obtain good statistics of aggregates in concentrated solutions. Nevertheless, we may note that in SiW LiTFSI solutions, ions are either free or form ion pairs, whereas WiS electrolytes are characterized by the appearance of larger aggregates, possibly occupying a large part of the system. To visualize structures formed by solvent and salt ions, we plotted in Figure 4 snapshots of selected MD frames for 1 and 20 m solutions, highlighting water molecules or ions; plots for 5 and 10 m are available in Figure S1 in the Supporting Information. In the SiW electrolyte, water molecules are in contact, and the water network spans the whole simulation box. Clearly, ions in this system are isolated and therefore interact almost exclusively with solvent molecules. Conversely, in the WiS electrolyte, large ion aggregates form structures penetrating the sample, whereas water molecules are isolated or form relatively small aggregates. Therefore, the amount of water–ion interactions is significantly increased. The structures obtained here from AIMD for 20 m solutions do not support the picture of water–salt domain separation;^8^ instead, they are consistent with the conclusions that water molecules in concentrated WiS electrolytes are rather isolated.^11,21^

**Figure 5:**
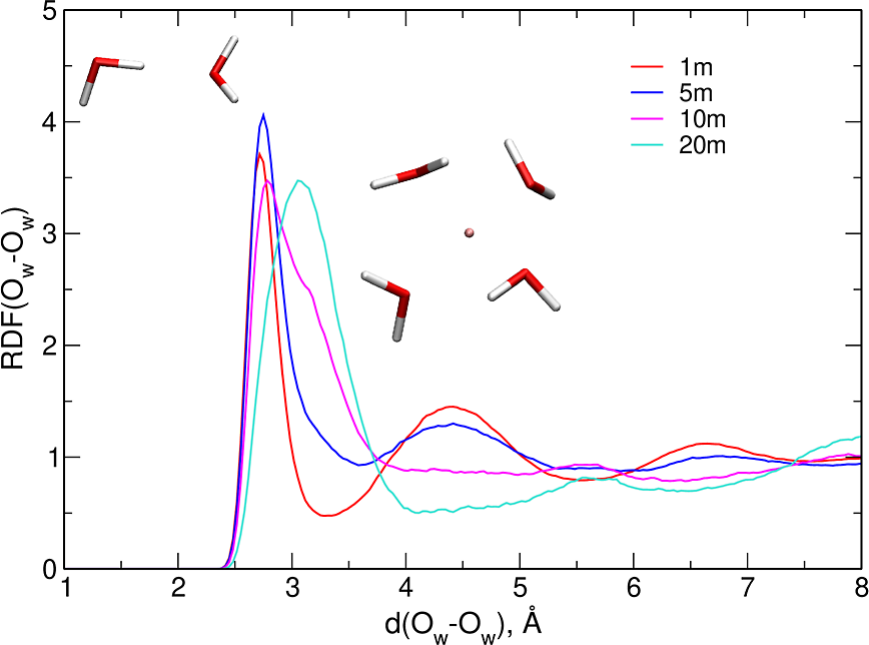
Radial distribution functions for the O_w_–O_w_ atom pairs and sample geometries contributing to the first maximum at low and high salt concentrations.

Looking for information about the structure of water–water interactions in the electrolytes, we calculated RDFs for the O_w_–O_w_ atom pairs, shown in Figure 5. At low LiTFSI concentrations, the first (and the highest) maximum in the RDF is located at 2.72–2.75 Å. It corresponds to an orientation of two water molecules typical for a tetrahedral water structure, that is, when one of the O–H bonds of one molecule is pointing toward the O atom of the other molecule, as schematically shown in the left part of Figure 5. The maximum at 2.78 Å is also the highest in the 10 m system, but a shoulder develops above 3 Å. In the most concentrated electrolyte, the first peak in the RDF is wider, and its maximum is shifted to 3.07 Å. Accompanying the shift in the position of the major peak are the changes at 4.5 and 6.7 Å – smaller peaks located at these distances in 1 and 5 m electrolytes vanish in the 10 m system, and the minima appear in the WiS 20 m electrolyte, indicating that the structure of water network is distorted from that observed at low concentrations. The RDF maximum above 3 Å can be attributed to the O–O distances between four or three water molecules solvating the central Li^+^ cation; a sample configuration extracted from the trajectory is shown on the right. Similar changes in the first maximum position in the O_w_–O_w_ RDF were reported from classical MD simulations;^11,13^ the effect obtained in ref (11) was smaller (a shoulder at 20 m), and our AIMD results are close to the findings of ref (13).

Figure S2 in the Supporting Information shows the RDFs and CNs for H–O interactions. The height of the first maximum in the H–O_w_ RDF, located between 1.75 and 1.84 Å, decreases with molality. An opposite trend is observed for the H–O_a_ RDF, where the maximum appears at a slightly larger distance of about 1.9 Å. Integrated H–O_w_ RDF for the 1 m system yields at a distance of 2.5 Å CN 0.94, suggesting that almost all hydrogen atoms are near water oxygen atoms. H-water CNs decrease with salt concentration, and in the 20 m solution, hydrogen atoms have only 0.2 O_w_ atoms on average. CNs for H-anion interactions increase in the same order; in the most concentrated electrolyte, hydrogen atoms have about 0.6 O_a_ atoms from TFSI anions within a distance of 2.5 Å. Nonsurprisingly, an increase of salt concentration lowers the probability of water–water HBs and increases the possibility of forming water–anion bonds.

To check whether the structures of the systems were changed during the AIMD simulations or if they primarily resulted from the force field-based simulations, we compared in Figure S3 in the Supporting Information the RDFs and integrated RDFs for Li–O atom pairs obtained in the classical and ab initio MD. A similar plot of the O_w_–O_w_ RDFs is presented as Figure S4. At all salt concentrations, positions of the first and second maxima in the Li–O_a_ and Li–O_w_ RDFs in the AIMD results appear at distances smaller than those in the classical simulations. Accordingly, there are differences in the calculated CNs. The Li–O_w_ CNs decrease in the AIMD results for 1–10 m solutions. The effect is the opposite for the Li–O_a_ CNs, where the largest difference is observed for the 10 m electrolyte. Above the distance of 3.5 Å, changes in Li–O CNs are observed at all molalities. Differences between classical and ab initio simulations are also noticeable in the case of the O_w_–O_w_ RDFs (Figure S4). The location of the first maximum is the most affected in the 20 m solution. On the other hand, the largest differences in the range 4–5 Å appear for small salt concentrations. These findings show that indeed there was a structure reorganization in the course of AIMD simulations, despite their short time.

**Figure 6:**
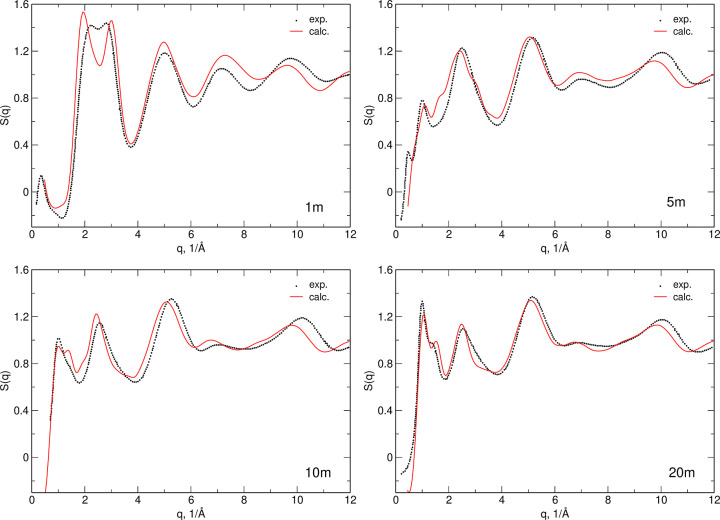
Calculated and experimental X-ray structure factors of aqueous LiTFSI electrolytes. Experimental data from ref (11).

As a test of the correctness of the structures obtained in the AIMD simulations, we computed X-ray structure factors S(*q*) using the ISAACS software.^33^ In Figure 6, calculated structure factors are compared to the experimental data from ref (11). The differences between simulations and experiment are more pronounced at low concentrations, and the most remarkable is the increased splitting of the two peaks at 2–3 Å^–1^ in the 1 m electrolyte. Reproduction of the experimental structure factor improves with increasing salt concentration. Overall, there is an agreement in the number of peaks and their positions, and the trends of the changes observed upon increasing LiTFSI concentration are well reproduced. These results indicate that the final AIMD trajectories adequately describe the structure of electrolytes; therefore, we proceeded to the analysis of HBs and the IR spectra.

**Figure 7:**
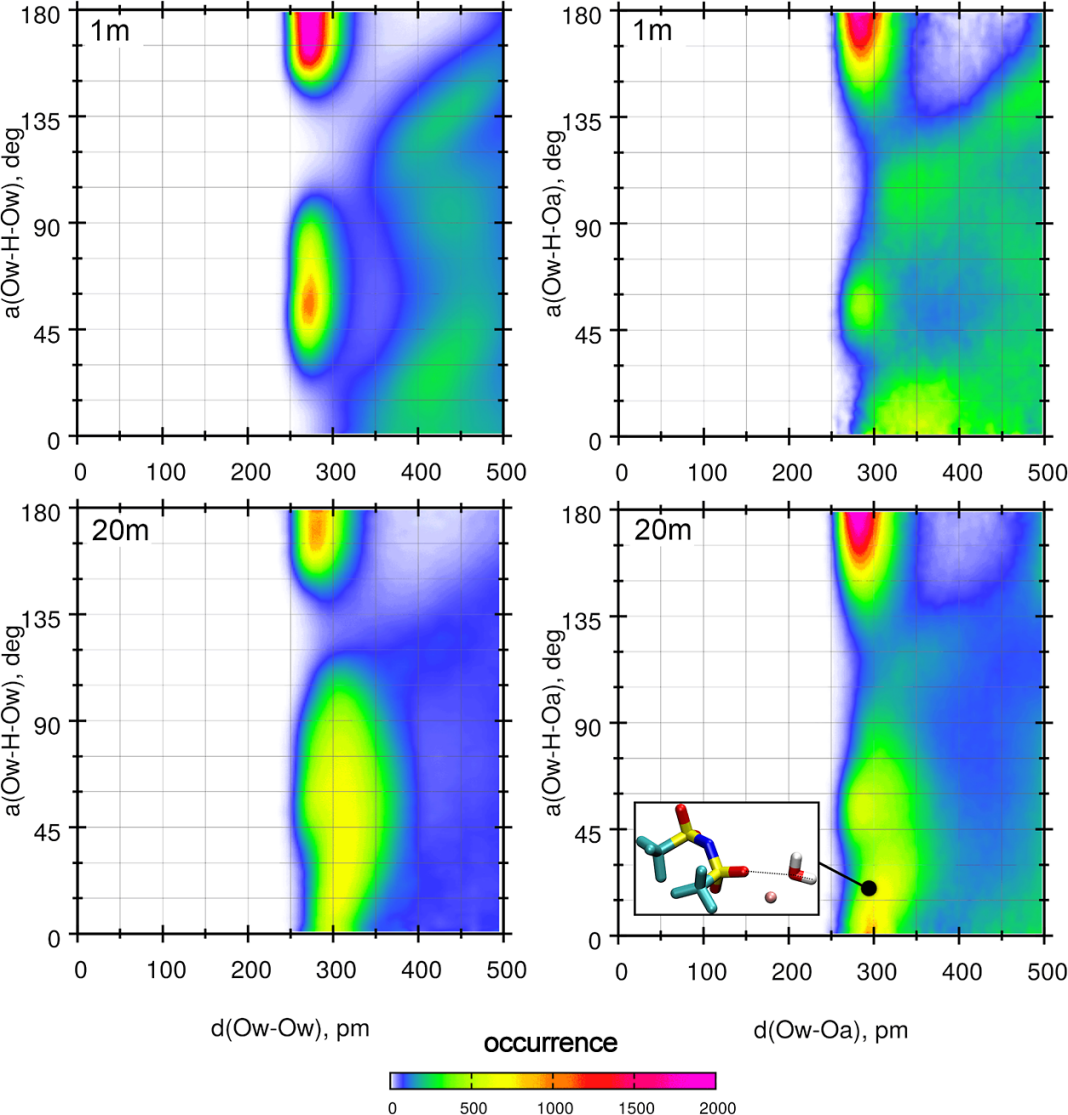
Combined distribution functions for water–water and water–anion structures.

For efficient hydrogen bonding, not only is a sufficiently small hydrogen-to-acceptor distance required but also the arrangement of D-H···A atoms (where D and A denote the donor and acceptor of the hydrogen atom, respectively) should be close to linear. Therefore, in Figure 7, we show combined distribution functions (CDFs) of D–A distances and D-H···A angles for water–water and water–anion pairs. The limits for possible HB formation correspond to distances less than 350 pm and angles close to 180°. A well-noticeable maximum of probability appears in this region in the CDF plot for water–water HBs in the 1 m solution, corresponding to a typical configuration in tetrahedral water structure (cf. the geometry shown in Figure 5). The lower maximum at the same D–A distance observed for angles between 45 and 60° is related to the other hydrogen atom of the donor water molecule. In the WiS electrolyte, the main maximum, corresponding to HB formation, is lower but still well pronounced. Simultaneously, there is an increase in the probability of configurations with D–A distances of about 300 pm and angles close to 0°. These arrangements, ineffective for hydrogen bonding, arise in the water structure that is broken by Li^+^ cations. According to the CDFs for water–anion interactions, exhibiting a pronounced maximum in the ranges of 280–300 pm and 150–180°, conditions for water-TFSI HBs are fulfilled at both concentrations shown in Figure 7. Again, in the 20 m electrolyte, the probability of small angles increases at 300 pm, and these geometries correspond to water-Li^+^-anion configurations similar to the example displayed in the inset of Figure 7.

**Figure 8:**
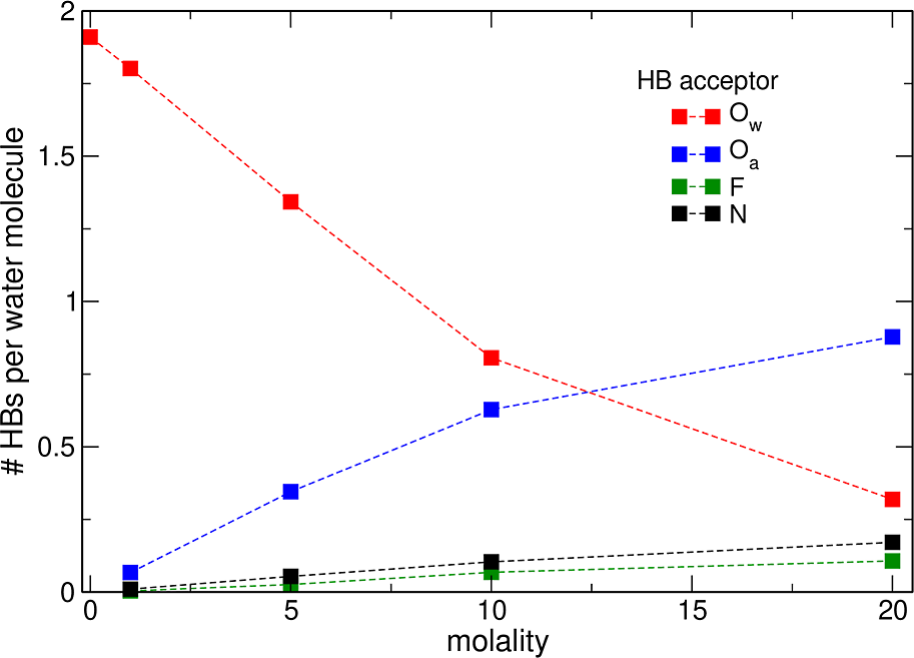
Average numbers of hydrogen bonds per water molecule at different salt concentrations.

RDFs and CDFs indicate that water–water and water–TFSI HBs are formed in the studied electrolytes. We obtained statistics of HBs for all samples, using the criteria as in our previous works,^22,34^ that is, the D–A distance less than 3.5 Å and the deviation of D–H···A from linearity by no more than 40°. In Figure 8, we present the average number of HBs per donor (water molecule) in a breakdown into different acceptor atoms. A similar plot of the average number of HBs per accepting molecule or anion is shown in Figure S5 in the Supporting Information. In our recent work, we calculated that the average number of HBs in neat water is 1.91 per H_2_O molecule.^22^ With increasing LiTFSI concentration, these bonds become less frequent, and in the 20 m electrolyte, there are only 0.3 O_w_–H···O_w_ bonds per H_2_O molecule. Simultaneously, the number of HBs to TFSI anions increases; most of these bonds are to the O_a_ acceptor (0.88 HB per donating water in the 20 m solution). The abundance of HBs to N and F acceptors is lower; nevertheless, in the WiS 20 m system, there are on average 0.47 and 0.3 HBs to N and F atoms, respectively. Although water–anion bonds partially replaced the water–water HBs in salt solutions, the total number of HBs per water molecule decreased from 1.88 in the 1 m solution to 1.47 in the 20 m electrolyte. This effect is a consequence of increasing water coordination with Li^+^ ions. Figure S5 shows that the average number of O_w_–H···O_a_ HBs per anion in 1 and 5 m solutions approaches four; therefore, almost all O atoms of TFSI ions are involved in HBs with water molecules as donors. This is possible because the Li^+^ concentration and, accordingly, Li–O_a_ CNs are small in these systems. The number of HBs per O_a_ atom decreases at higher LiTFSI concentrations, where there are fewer water molecules, and the Li-anion coordination increases. Interestingly, the average number of HBs per accepting F or N atom is not affected significantly by the salt concentration, from which we may deduce that the reason for decreased number of HBs to anion oxygen atoms is not that much the reduced availability of water molecules but rather the competition between hydrogen bonding and Li–O_a_ coordination.

### 3.2 IR Spectra

In this section, we will analyze the relation between the structure of LiTFSI/water electrolytes and their vibrational spectra. The simulated IR spectra in the range 0–4000 cm^–1^ are shown in Figure S6 in the Supporting Information; the calculated spectrum of neat water was taken from ref (22). An intense band of stretches of the O–H bond appears above 3000 cm^–1^. Three bands between 900 and 1300 cm^–1^ are the oscillations of the TFSI anions. Li–anion interactions can affect the frequencies of S=O bond stretching. In ref (9), changes in the corresponding band at 1330–1350 cm^–1^ were observed for increasing molality of the electrolyte: the normalized intensity of the band decreased, and the contribution from a component split into two maxima increased. In our AIMD-simulated spectra, the S=O stretching vibration is shifted to 1290 cm^–1^, that is, to a lower frequency than that measured experimentally. A plot of calculated IR intensity in this spectral region, normalized to the LiTFSI concentration, is shown in Figure S7 in the Supporting Information. Unfortunately, the resolution of our spectra, obtained for small systems and short simulation time, is too crude to monitor changes in the band shape, and only the decrease in the band intensity is noticeable, in agreement with the experiment.

**Figure 9:**
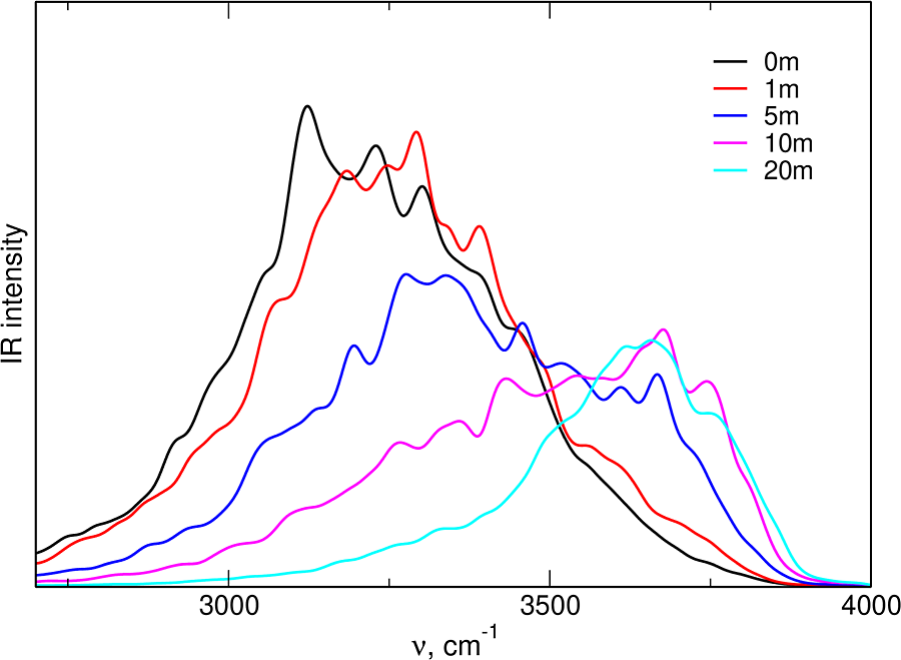
IR spectra of aqueous LiTFSI electrolytes in the range of the O–H stretching frequency, calculated from AIMD simulations.

Therefore, we focus on the region of stretching vibrations of water molecules where the most prominent changes in the spectra are observed. The spectra are shown in Figure 9. It can be readily seen that the IR intensity shifts to higher energies upon an increase in the electrolyte concentration. The maximum obtained for neat water at 3150–3200 cm^–1^ decreases, and a new band gradually develops at 3650–3700 cm^–1^. Calculated spectra are in good agreement with the experimental results from refs (11),^12^.

The features observed in the LiTFSI/water electrolytes at 3000–4000 cm^–1^ are most naturally attributed to the changes in the environment of water molecules and the pattern of HBs. To analyze the relation between the local structure and the spectrum, we used FTs of O–H bond lengths to compute the frequencies of O–H stretching oscillations for all water molecules in the system. Figure S8 in the Supporting Information presents an example of the obtained power spectra. At both concentrations shown, the frequencies of oscillations of individual O–H bonds vary over a wide range. In the 1 m electrolyte, maxima for most frequencies appear between 3300 and 3500 cm^–1^, but there are also some O–H groups with vibrational frequencies close to 3000 cm^–1^ and a set of O–H bonds with stretching frequencies of about 3750 cm^–1^. In the 20 m solution, the distribution of the frequencies is shifted toward 3750–3800 cm^–1^; nevertheless, some O–H bonds oscillate with much lower frequencies of 3000–3300 cm^–1^. Apparently, the change in salt concentration results in a shift of the average O–H stretching frequency, following the change of the average structure of HBs. At all concentrations, there are few O–H groups with vibrational frequencies far from the average, indicating that the local environment of some water molecules is different.

**Figure 10:**
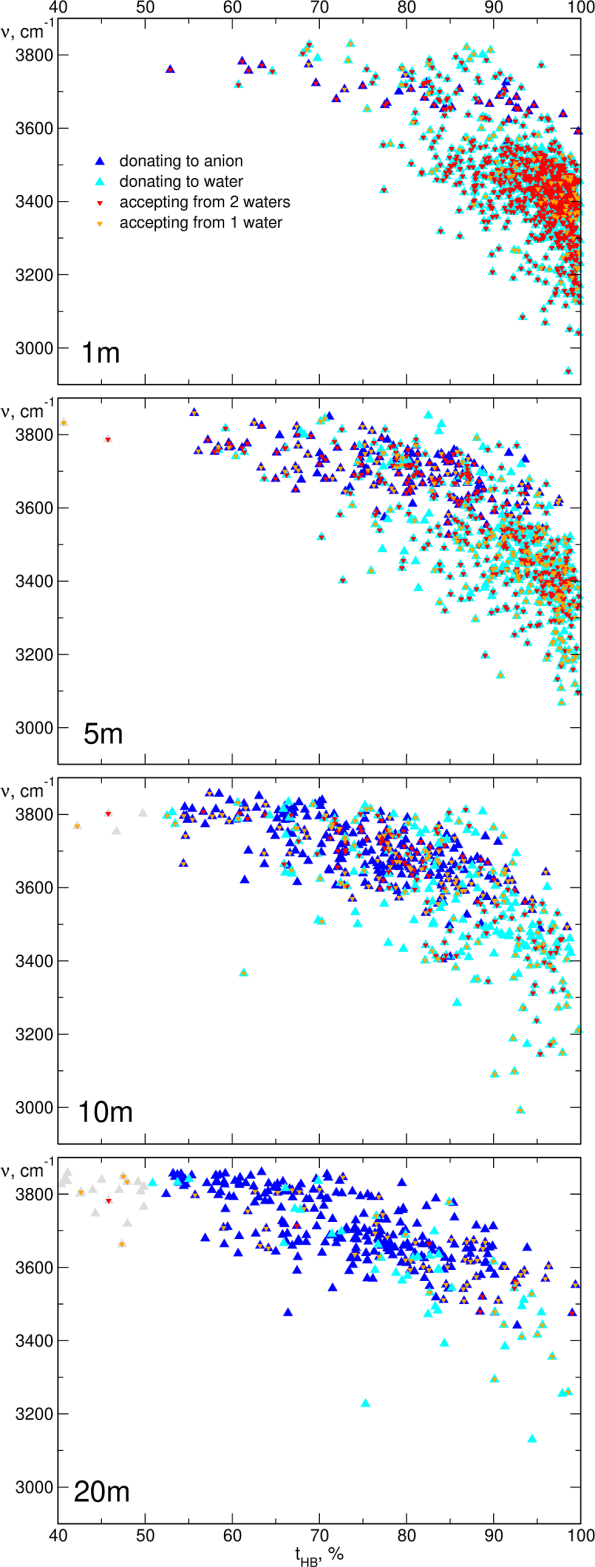
Positions of the maxima in FTs of the O–H bond lengths vs the time of HB formation for LiTFSI electrolytes.

In the next step, we calculated the frequencies of the maxima in the Fourier-transformed lengths of the O–H bonds and compared them to the average time of HB formation by a given water molecule. The results are shown in Figure 10. For O–H bonds involved in hydrogen bonding for more than 50% of the time, we marked whether the H atom is donated to an O_w_ or O_a_ acceptor. Additionally, we labeled whether the water molecule is an acceptor of one or two HBs from the other water molecules.

It is readily noticeable in Figure 10 that the stretching frequency of the O–H bond correlates with the time of HB formation: the highest frequencies are observed for the O–H groups participating in hydrogen bonding for short times; increasing the time of hydrogen donation shifts the oscillation to lower frequencies. The dependence is approximately linear for the O–H bonds donating the hydrogen to the O_a_ acceptors in 1 and 5 m solutions. There is a clear difference between O–H groups donating to an anion and to a water molecule. For the former, the frequencies are in the range of 3600–3800 cm^–1^; in the latter case, the frequency may decrease to 3000 cm^–1^. The O–H stretching frequency is significantly lowered when the water molecule acts not only as a hydrogen donor but also as a hydrogen acceptor. Comparing in Figure 10 plots for increasing LiTFSI concentration, one can easily observe how the average frequency of the O–H oscillations increases with the molality of the electrolyte. In the 1 m solution, most water molecules donate H atoms to water for almost all the simulation time, being simultaneously acceptors from other water molecules (tetrahedral water structure). Accordingly, vibrational frequencies for the majority of the O–H groups are in the range 3000–3400 cm^–1^. There are some H_2_O molecules participating in the HB to a TFSI anion, with stretching frequencies above 3600 cm^–1^, but at low concentrations, they do not contribute significantly to the spectrum. At 5 and 10 m, the average time of hydrogen bonding decreases as well as the probability that the water molecule is a hydrogen atom acceptor. Simultaneously, the number of O–H groups donating to O_a_ atoms increases. All of these factors lead to the increase in average stretching frequency. Finally, in the most concentrated 20 m system, most water molecules donate hydrogen to anions. Only few water molecules are hydrogen acceptors, usually from only one H_2_O molecule, because there is an insufficient amount of water molecules to form water–water HBs at high anion concentration. As a result, the O–H frequencies for most water molecules are in the range 3600–3800 cm^–1^. Analysis of individual O–H stretching frequencies and HB formation in Figure 10, together with the statistics of HBs shown in Figure 8, explains very well the changes in simulated IR spectra (Figure 9), which can undoubtedly be assigned to the changes of HBs formed by water molecules.

**Figure 11:**
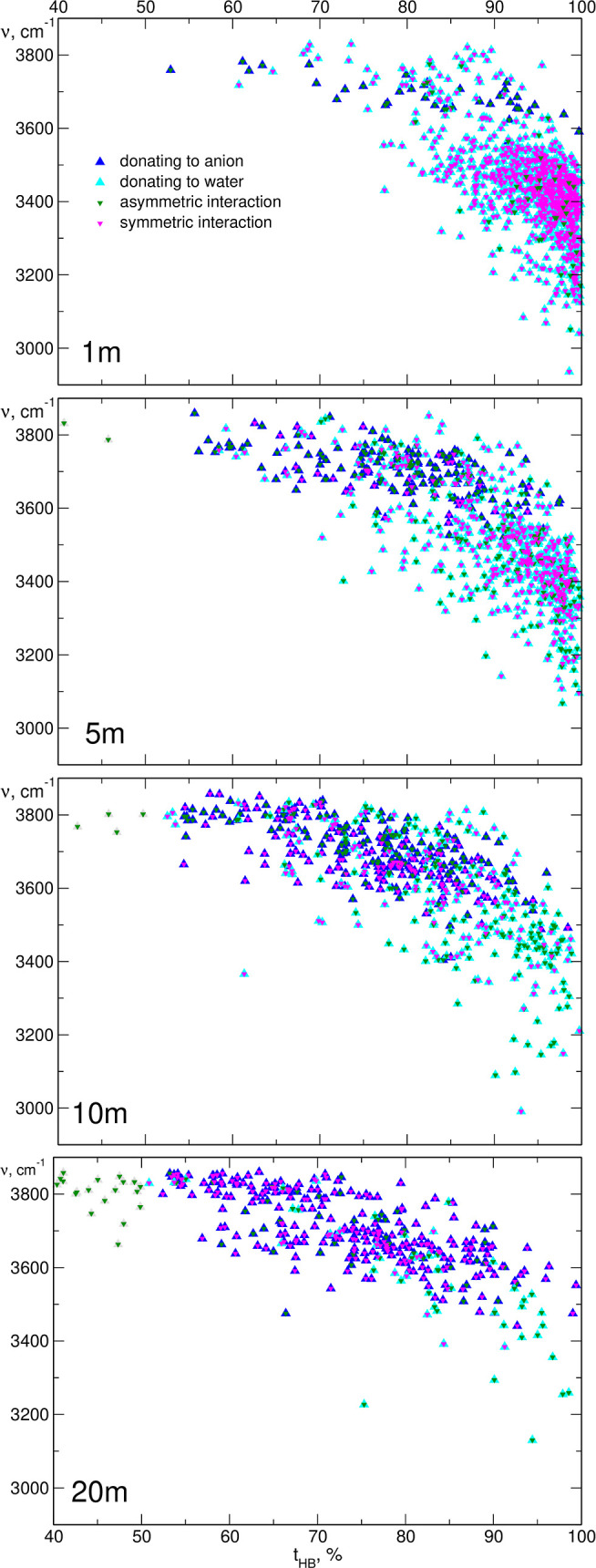
An alternative labeling of the data from Figure 9, indicating the symmetry of the local environment.

It is also interesting to analyze whether both O–H groups from a water molecule donate hydrogen atoms to two different (O_w_ and O_a_; asymmetric interaction) or two acceptors of the same kind (2O_a_ or 2O_w_; symmetric interaction). In Figure 11, we present the same data as in Figure 10, but with labels indicating the type of local environment. In the dilute 1 m solution, the majority of water molecules are in a symmetric environment, donating both hydrogens to the O_w_ acceptors. Only a few waters interact with the O_w_ and the O_a_ atoms; accordingly, all interactions with anion acceptors are marked as asymmetric. With increasing molality, the probability of water–anion interaction increases with a decrease in water–water bonding. Therefore, more H donation to water acceptor occurs in an asymmetric environment, whereas interactions with TFSI anions become symmetric. Finally, at a 20 m concentration, the situation is reversed compared to the 1 m solution—almost all interactions with water are asymmetric, and donation to O_a_ atoms is mostly symmetric.

**Figure 12:**
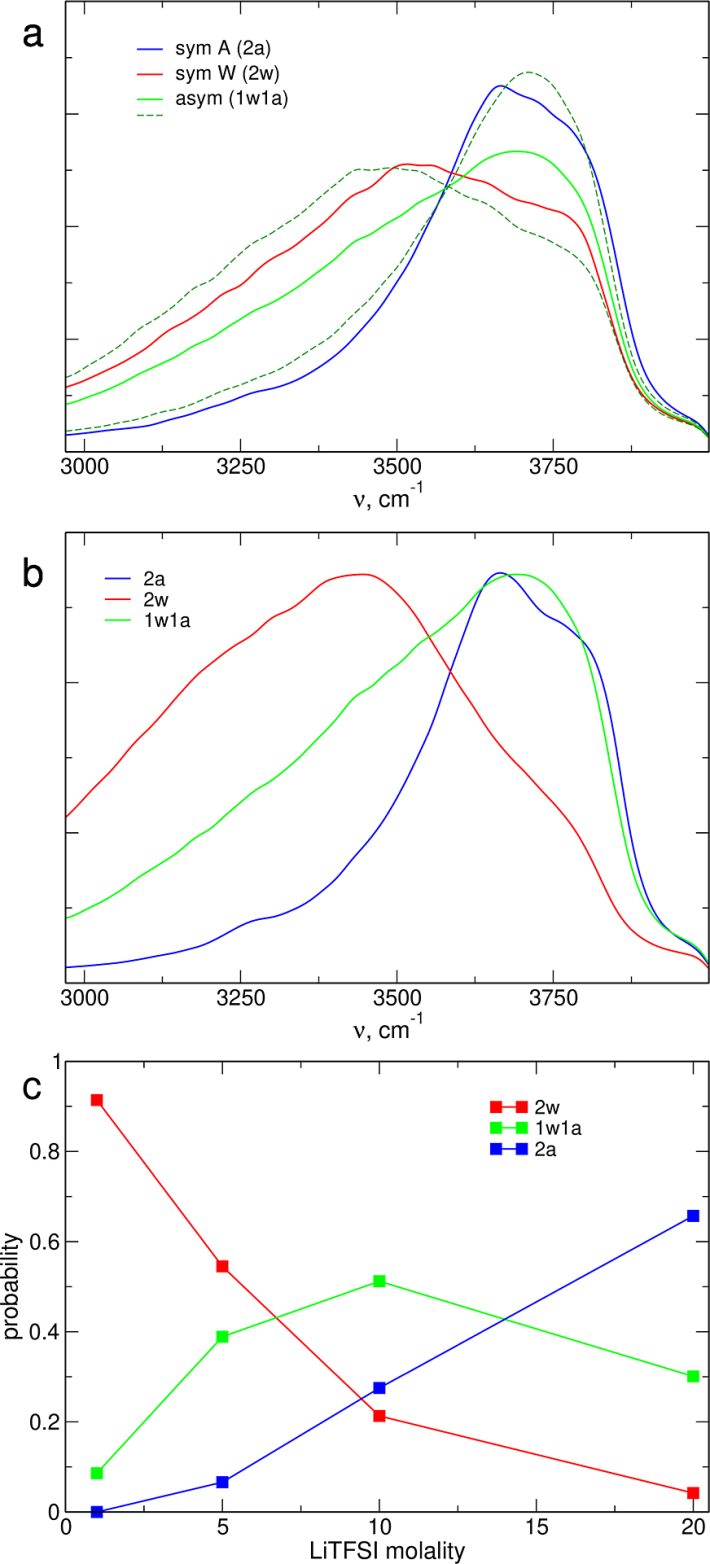
Average contributions to the power spectrum of the 10 m electrolyte (a). Normalized contributions of different environments, see text for details (b). Percentage of contributions vs salt molality (c).

Based on the configurations of HBs extracted from the AIMD trajectories and the FTs of O–H distances, we calculated the average contribution of each type of environment (following refs (11),^12^, labeled here as *2w*, *1w1a*, and *2a*) to the power spectrum of the 10 m electrolyte (Figure 12a). The maximum of the average power spectrum of the O–H bond interacting with TFSI anions in symmetric environments (*2a*) appears at about 3600 cm^–1^. The band for the symmetric interaction of the O–H groups with water molecules (*2w*) is broader, and the maximum is located at 3500 cm^–1^. Two bands (broken lines in Figure 12a) are presented for H_2_O molecules in the asymmetric environment, arising from the occurrence of the O–H groups engaged in HBs with the O_a_ or the O_w_ atoms. The maxima of these bands are close to the corresponding maxima for symmetric interactions, and their average produces the band labeled as *1w1a* (solid green line) with the maximum at about 3700 cm^–1^ and a long tail to lower frequencies.

The contributions to the power spectrum shown in Figure 12a were calculated for the 10 m electrolyte. As may be deduced from Figure 11, the size of each contribution and, to some extent, also its average frequency depend on the salt concentration. In Figure 12b, we combined the three components, calculated for the electrolytes in which they are the largest, that is, *2w* at 1 m, *2a* at 20 m, and *1w1a* at 10 m concentration (cf. Figure 12c). The components were normalized to the same intensity at the maximum. The *2w* contribution of symmetric interactions to water molecules is broad, with the maximum at 3450 cm^–1^. The contribution arising from asymmetric *1w1a* interactions exhibits strong asymmetry, with a long tail at the low-frequency side and a maximum at 3700 cm^–1^. The symmetric interactions with the anion (*2a*) produce a narrower band with a splitting of about 100 cm^–1^. Figure 12b can be compared to Figure 9a from ref (11), where the same contributions extracted from the IR spectra are shown. There is a striking similarity between these two plots, confirming that the DFT-based AIMD simulations give a good description of the HBs formed in aqueous LiTFSI solutions. Some differences between the experiment and our analysis can be attributed to the differences in the observed and calculated frequencies. It should also be noted that the decomposition in ref (11) was based on IR intensity, whereas our analysis used the power spectrum. According to the results of ref (21), this will affect mainly the *2w* component, because for the WiS electrolyte, the difference between the IR and power spectrum above 3000 cm^–1^ was small.

Figure 12c presents the changes in the abundance of different hydrogen bonding environments of O–H groups upon increasing the electrolyte concentration. The percentage of *2a* and *2w* reflects the amount of anion or water HB acceptors in the system: the former increases and the latter decreases with increasing salt molality. At a 10 m concentration, populations of both kinds of interactions become approximately equal (21% of *2w* and 28% of *2a*). In the 1 m electrolyte, the symmetric *2a* interactions are almost absent, in agreement with Figure 11, showing that all interactions with anions are in an asymmetric environment (91%). Accordingly, the *1w1a* population at 1 m is about 9%; the contribution of asymmetric interactions increases with salt content up to 10 m, at which the concentration reaches a maximum (51%) and exceeds the abundance of both symmetric environments. Then, the *1w1a* percentage decreases slowly, but at 20 m, it still amounts to 30%. At this concentration, the *2a* component dominates with a contribution of 66%. The data in Figure 12c agree quite well with the probabilities obtained in ref (11) from the decomposition of experimental spectra (Figure 9c in ref (11)). The most significant differences are observed for the 20 m WiS electrolyte, in which experimental contributions *1w1a* and *2a* are approximately equal, whereas our results of water HBs predict a much larger probability of the asymmetric *1w1a* environment. On the other hand, at 10 m, the experimental *1w1a* probability is about 10% larger than that obtained from AIMD.

To underline the most important points of our results, we note that the AIMD simulations satisfactorily reproduced the structure of studied electrolytes, as indicated by the agreement of calculated structure factors, IR spectra in the range of the O–H stretches, and the contributions of different water environments to the experimental data. Therefore, we expect that the data on the coordination of Li^+^ ions, statistics of the HBs, and their local structure around water molecules extracted from our simulations provide a reliable description of real systems. These parameters are related to the mobility of ions and molecules relevant to the transport properties of the electrolyte. Although the time scale of the AIMD simulations is not sufficient for calculations of the latter, the AIMD data are useful as a reference for other approaches (force field or machine learning).

## 4 Conclusions

We performed AIMD simulations for a series of LiTFSI solutions in water, changing salt concentrations from 1 m (SiW electrolyte) to 20 m (WiS system). The analysis of the structure of electrolytes revealed that Li^+^ cations are preferably coordinated to water molecules, and interactions with anions become important only at sufficiently high LiTFSI molality. In dilute solutions, salt ions are solvated by water, but in WiS electrolytes, they form aggregates. Conversely, the network formed by water molecules is disrupted by salt ions, and at the highest concentration, water molecules are isolated or associate into small clusters. These findings confirm the results of recent classical MD simulations^11,12^ and agree with an AIMD study employing the BLYP functional.^21^

Statistics of different types of hydrogen bonds formed in the solutions showed that the water–anion interactions replace the water–water HBs when the molality of the electrolyte increases but the total number of HBs decreases in WiS systems. These changes in the interactions lead to pronounced changes in the IR spectra in the region of water stretching vibrations. The IR spectra simulated from the AIMD trajectories are in good agreement with the experimental results. Analysis of local configurations of HBs formed by water molecules allowed us to correlate the shifts of the IR intensity with the changes in the hydrogen bonding pattern. In SiW solutions, most of the O–H groups interact with other water molecules in a symmetric environment. Interactions with anions are less probable because of an insufficient amount of anion acceptors and occur in an asymmetric environment. These proportions are reversed in WiS electrolytes, where most water molecules interact symmetrically with TFSI oxygen atoms and the less abundant HBs to water acceptors are in asymmetric configurations. Contributions from different types of local environments and their abundance extracted from the AIMD results agree well with the decomposition of experimental IR spectra.^11^ This result corroborates not only the correct reproduction of the structure of the electrolyte but also the usefulness of our method of extracting the frequencies of local vibrations, through *a posteriori* analysis of a MD trajectory based on FTs of selected distances.

The overall agreement of the AIMD results reported here with the experimental data confirms the applicability of AIMD simulations based on the density functional methodology to the WiS electrolytes. The high cost of ab initio calculations limits the size of the systems and the time scale of simulations. Therefore, the prospective way of extending the investigations on WiS electrolytes is to develop a machine-learned force field, allowing an increase in the size of simulations while maintaining the ab initio accuracy.

## Supporting Information Available

Snapshots of selected frames from 5 and 10 m electrolytes, RDFs for H–O atom pairs, comparison of RDFs from classical and ab initio MD, average numbers of HBs per acceptor, calculated IR spectra in the full range and in the region of S=O vibrations, FTs of O–H bond lengths, and sample input and parameter files (PDF)
