# Supplementary material for: Ab Initio Molecular Dynamics Simulations of Aqueous LiTFSI Solutions—Structure, Hydrogen Bonding, and IR Spectra
Source: J Phys Chem B. 2024 Jan 18;128(4):1001–11. doi: 10.1021/acs.jpcb.3c06633 (PMC10839825; doi:10.1021/acs.jpcb.3c06633)
Supplement: Supplementary file 1 — jp3c06633_si_001.pdf [file jp3c06633_si_001.pdf]

Ab Initio Molecular Dynamics Simulations of Aqueous LiTFSI Solutions – Structure, Hydrogen Bonding and IR Spectra

Piotr Wróbel, Piotr Kubisiak, Andrzej Eilmes*

Faculty of Chemistry, Jagiellonian University, Gronostajowa 2, 30-387 Kraków, Poland

Supporting Information

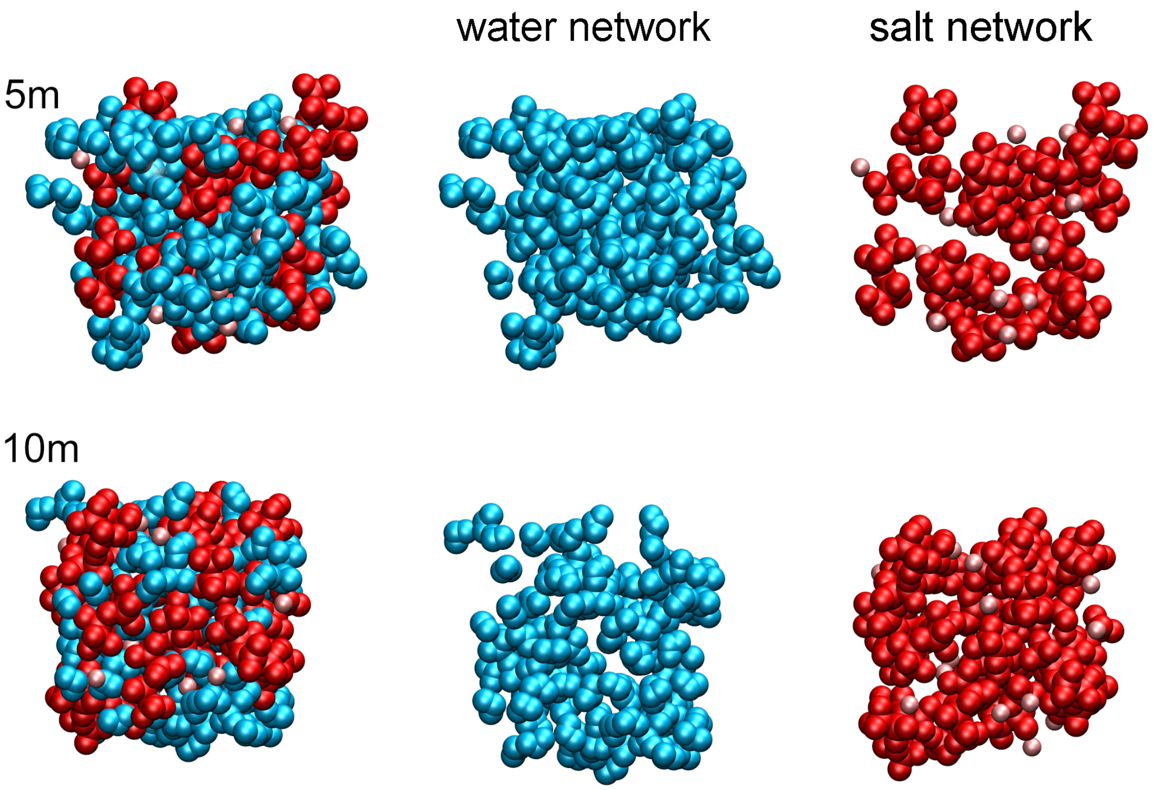

Figure S1. Snapshots of selected frames from the AIMD trajectories for 5m and 10m LiTFSI/water solutions with a decomposition into water and salt network. Water molecules – blue, TFSI anions – red, Li cations – pink.

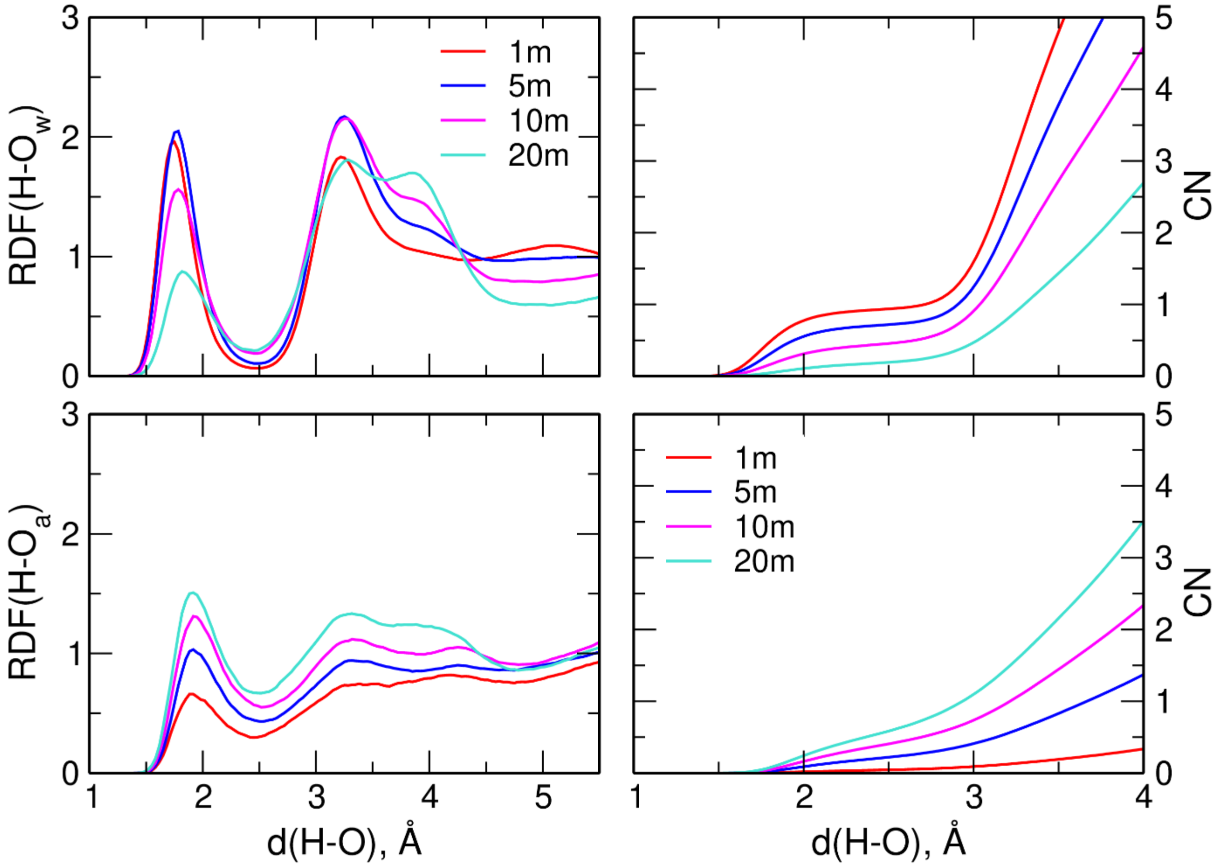

Figure S2. Radial distribution functions and integrated RDFs for H-O$_w$ and H-O$_a$ atom pairs in LiTFSI/water electrolytes.

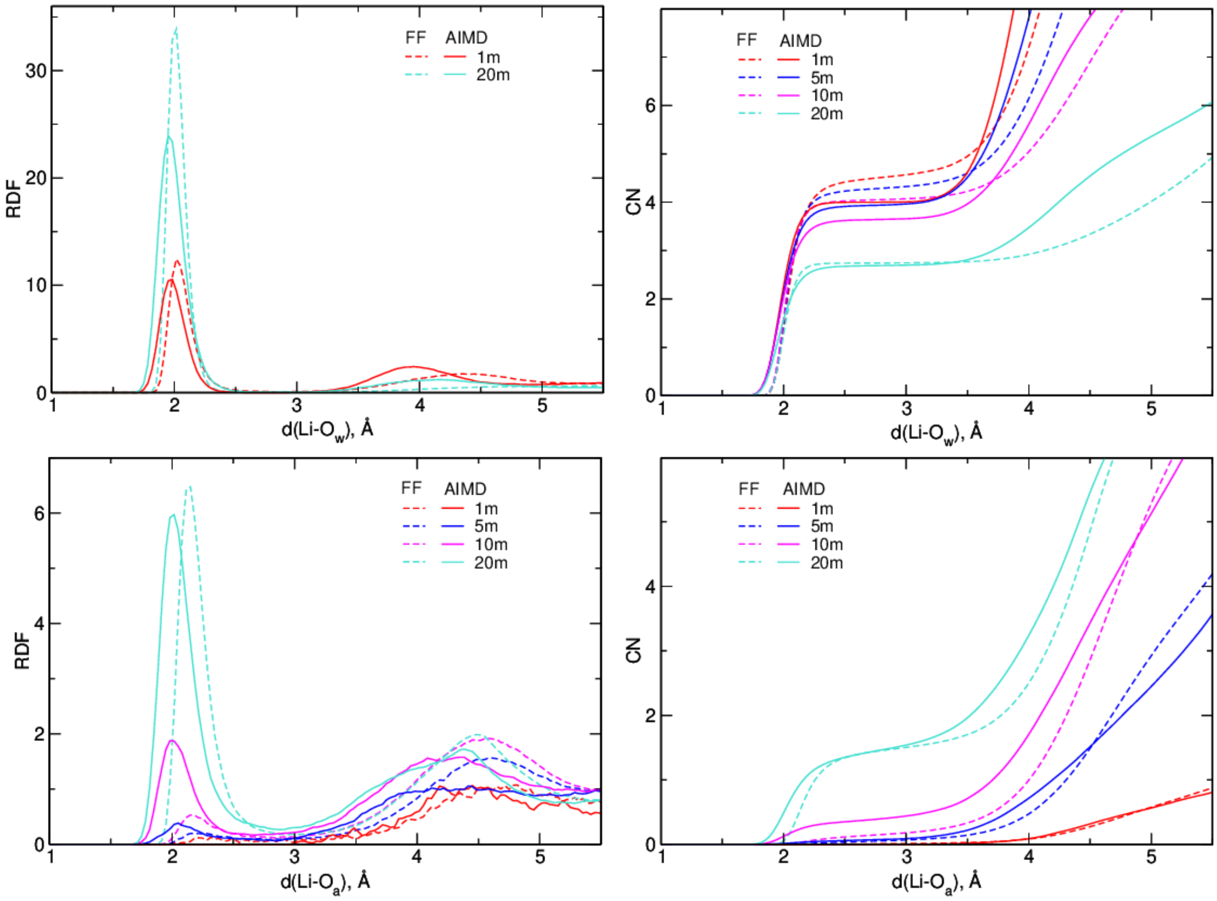

Figure S3. RDFs and integrated RDFs for Li-O_w and Li-O_a atom pairs obtained from classical (FF) and ab initio (AIMD) MD simulations.

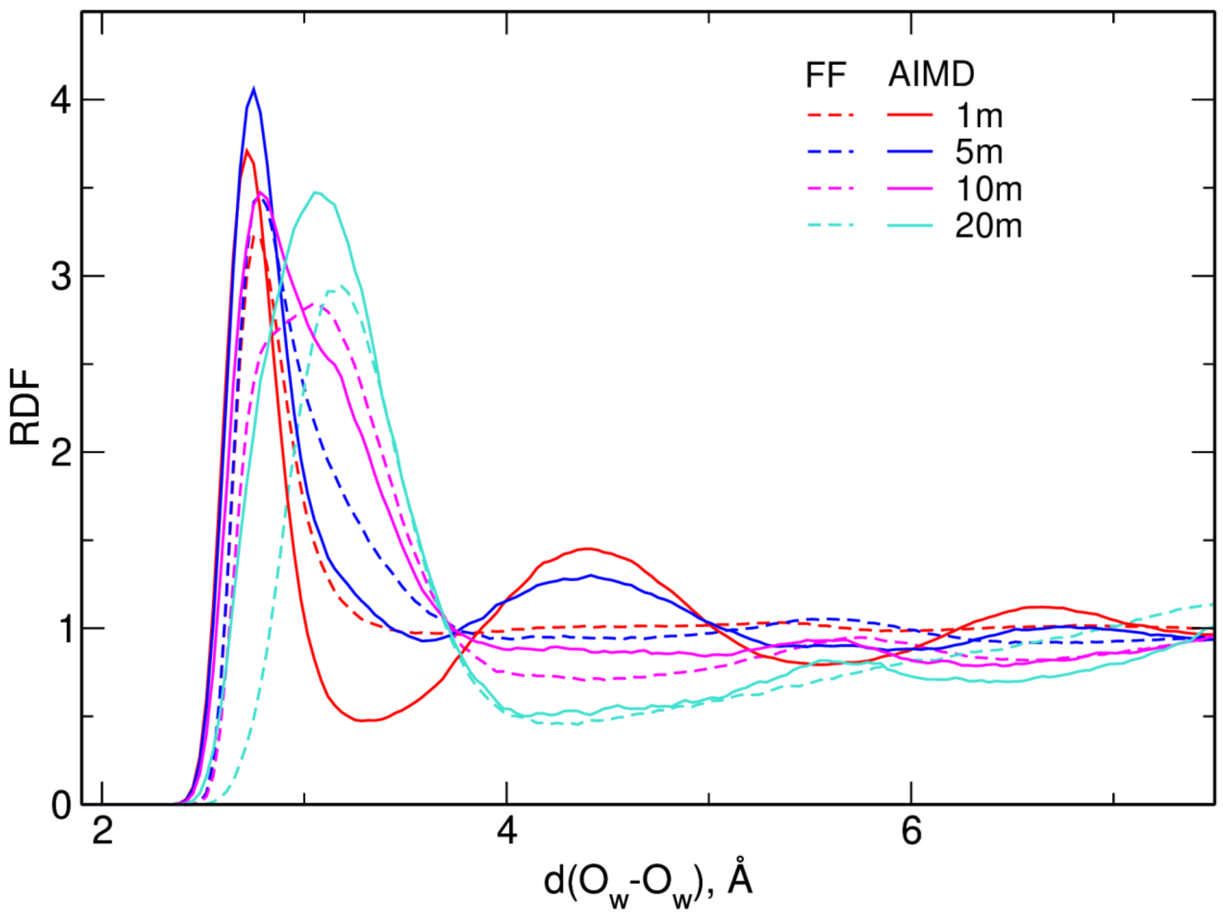

Figure S4. Radial distribution functions for O_w-O_w atom pairs obtained from classical (FF) and ab initio (AIMD) MD simulations.

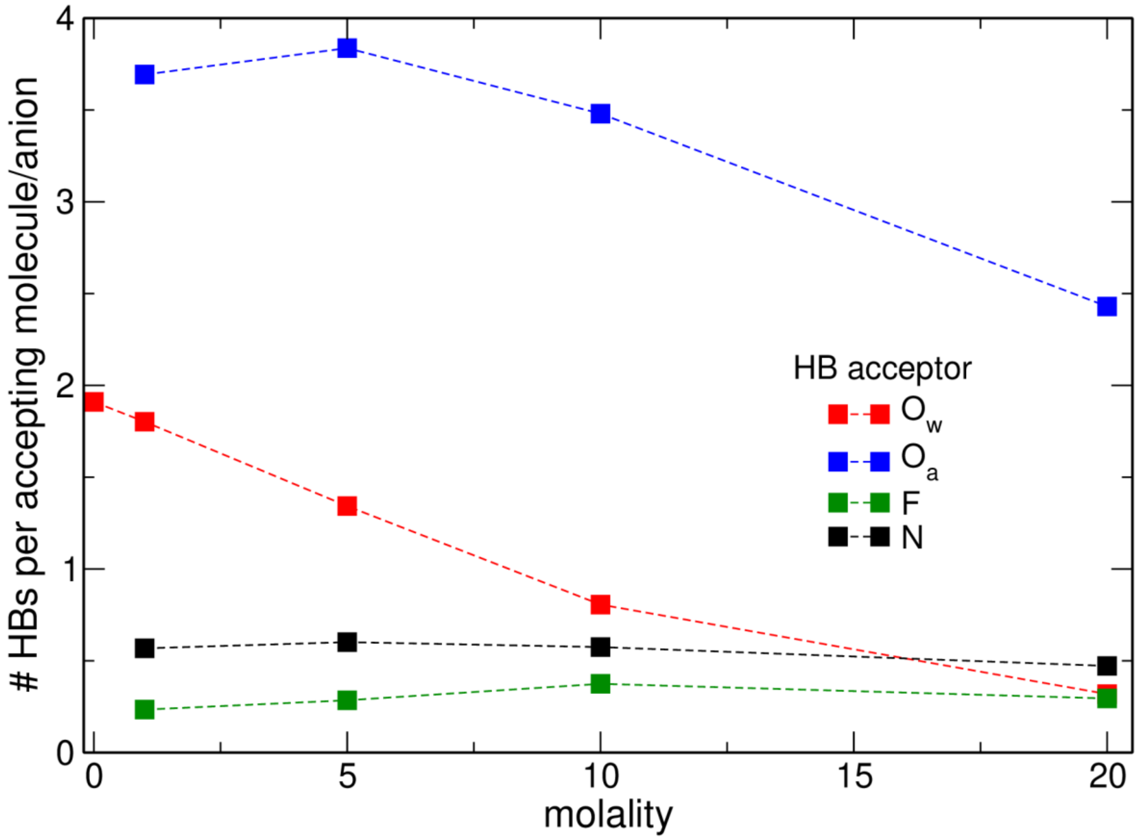

Figure S5. Average numbers of hydrogen bonds per accepting molecule or anion at different concentrations.

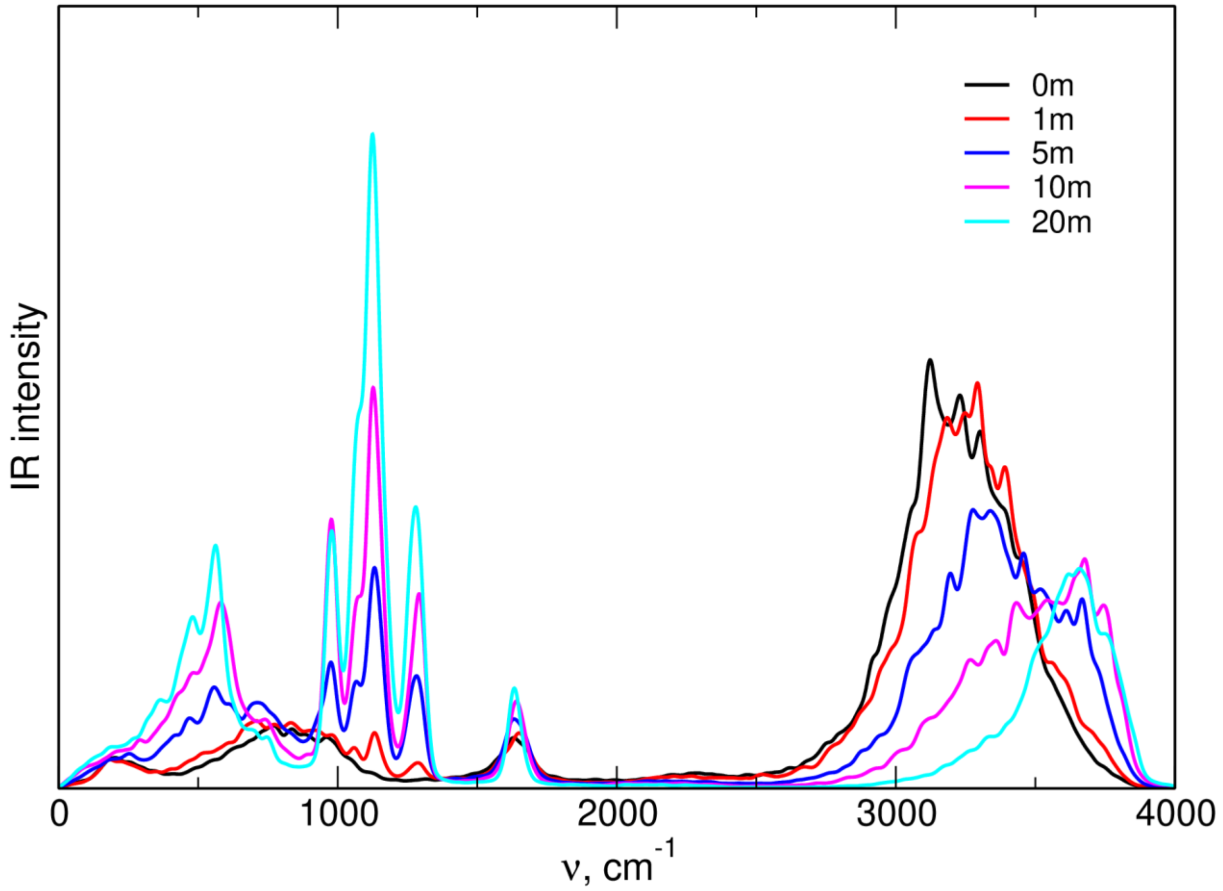

Figure S6. IR spectra obtained from AIMD simulations for LiTFSI/water electrolytes.

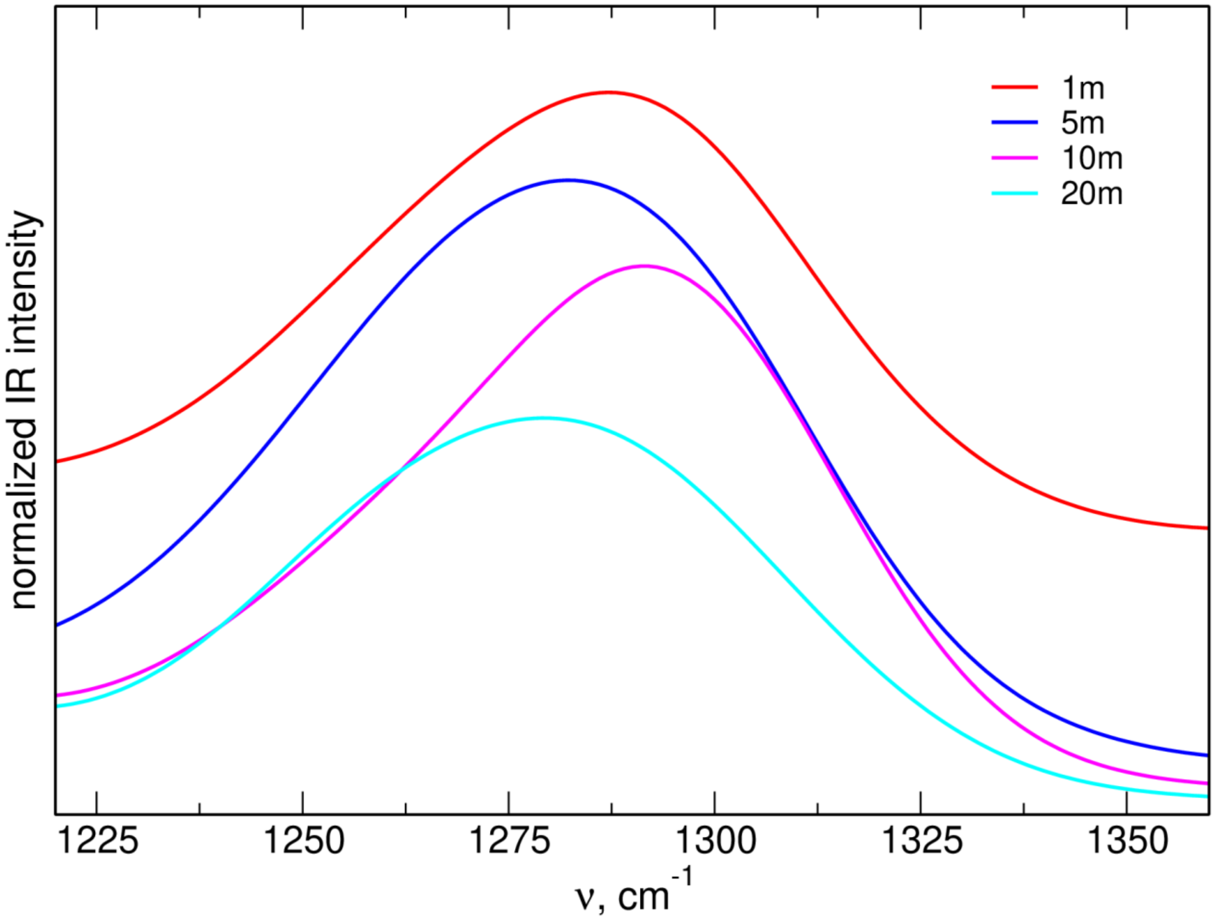

Figure S7. IR spectra obtained for LiTFSI/water systems from AIMD simulations in the region of S=O vibrations. Intensity has been normalized to the salt concentration.

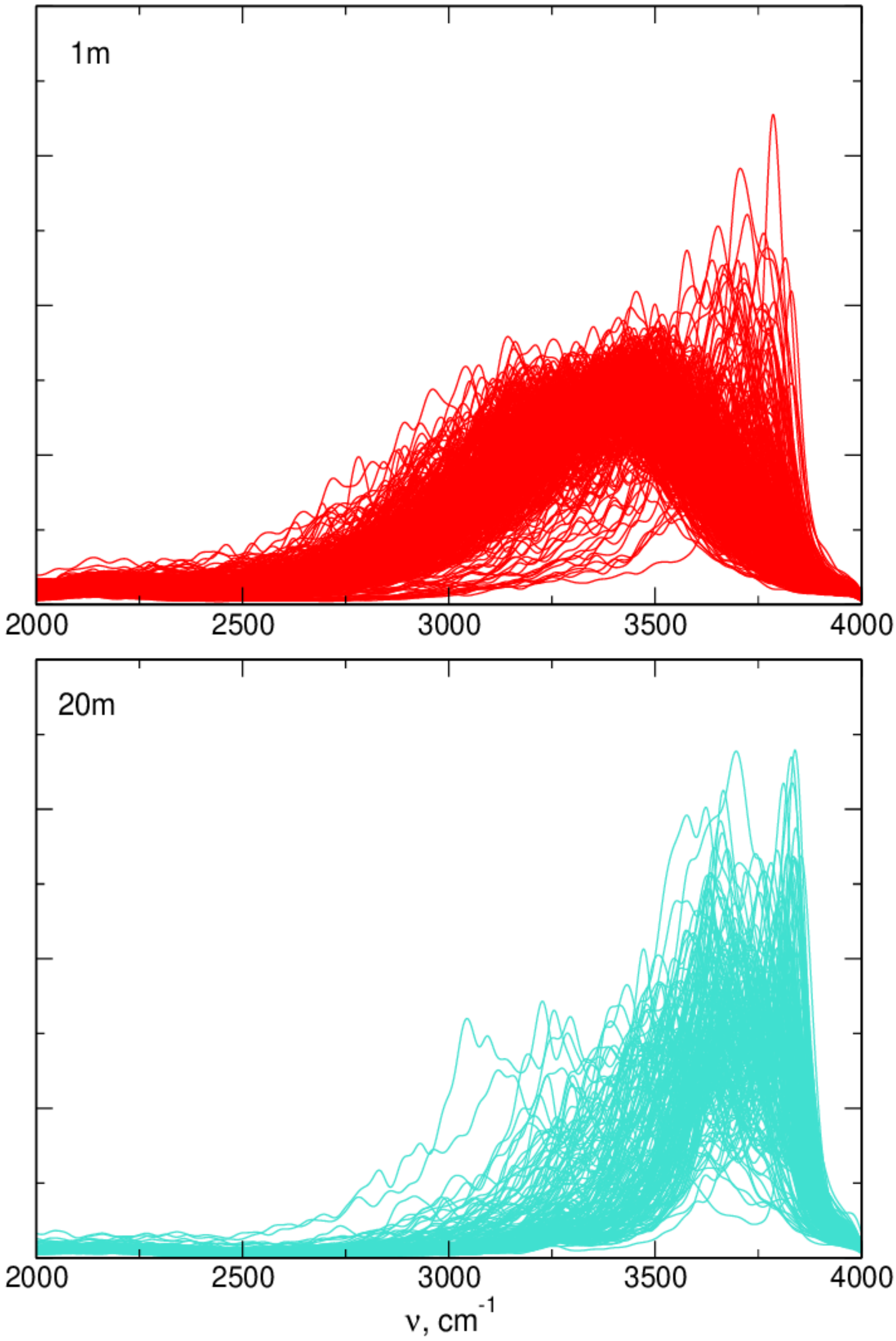

Figure S8. Fourier transforms of O-H bond lengths for all water molecules in the systems with 1m and 20m LiTFSI concentrations.

Appendix

Classical force field parameters in the NAMD format.

```
BONDS
!!!!!!!!!!!!!!!!!!!!!!!!!!!!!!!!!!!!
! ********* V(bond) = Kb(b - b0)**2
!
!Kb: kcal/mole/A**2
!b0: A
!
!atom type Kb   b0
!!!!!!!!!!!!!!!!!!!!!!!!!!!!!!!!!!!!
!! TFSI LOPES/PADUA
Ct        Ft        441.8       1.323
St        Ct        235.4       1.818
St        Ot        637.1       1.442
Nt        St        372.0       1.570
!!TIP3P
OW        HW        450.0       0.9572

ANGLES
!!!!!!!!!!!!!!!!!!!!!!!!!!!!!!!!!!!!!!!!!!!!!!!!
! ********* V(angle) = Ktheta(Theta - Theta0)**2
!
!V(Urey-Bradley) = Kub(S - S0)**2
!
!Ktheta: kcal/mole/rad**2
!Theta0: degrees
!Kub: kcal/mole/A**2 (Urey-Bradley)
!S0: A
!
!atom types     Ktheta    Theta0   Kub     S0
!!!!!!!!!!!!!!!!!!!!!!!!!!!!!!!!!!!!!!!!!!!!!!!!!
!! TFSI LOPES/PADUA
Ft       Ct       Ft          93.3      107.1
St       Ct       Ft          82.9      111.8
Ct       St       Ot         104.0      102.6
Ot       St       Ot         115.8      118.5
Ot       St       Nt          94.3      113.6
Ct       St       Nt          97.5      100.2
St       Nt       St          80.2      125.6
!!TIP3P
HW       OW       HW          55.0      104.52


DIHEDRALS
!!!!!!!!!!!!!!!!!!!!!!!!!!!!!!!!!!!!!!!!!!!!!!!!!!!!!!
! ******** V(dihedral) = Kchi(1 + cos(n(chi) - delta))
!
!Kchi: kcal/mole
!n: multiplicity
!delta: degrees
!
!atom types             Kchi    n   delta
!!!!!!!!!!!!!!!!!!!!!!!!!!!!!!!!!!!!!!!!!!!!!!!!!!!!!!
!! TFSI LOPES/PADUA
Ft       Ct       St       Ot           0.173     3      0.0
St       Nt       St       Ot          -0.0018    3      0.0
Ft       Ct       St       Nt           0.158     3      0.0
St       Nt       St       Ct           3.916     1      0.0
```

```
St       Nt       St       Ct           -1.245     2    180.0
St       Nt       St       Ct           -0.382     3      0.0

NONBONDED  NBXMOD 5  ATOM CDIEL FSHIFT VATOM VDISTANCE VFSWITCH -
     CUTNB 14.0  CTOFNB 12.0  CTONNB 10.0  EPS 1.0  E14FAC 0.5  WMIN 1.5

!!!!!!!!!!!!!!!!!!!!!!!!!!!!!!!!!!!!!!!!!!!!!!!!!!!!!!!!!!!!!!!!!!!!!!!!!!!
!!!!!!
!********* V(Lennard-Jones) = Eps,i,j[(Rmin,i,j/ri,j)**12 -
2(Rmin,i,j/ri,j)**6]
!
!epsilon: kcal/mole, Eps,i,j = sqrt(eps,i * eps,j)
!Rmin/2: A, Rmin,i,j = Rmin/2,i + Rmin/2,j
!
!atom  ignored    epsilon      Rmin/2   ignored   eps,1-4       Rmin/2,1-4
!!!!!!!!!!!!!!!!!!!!!!!!!!!!!!!!!!!!!!!!!!!!!!!!!!!!!!!!!!!!!!!!!!!!!!!!!!!
!!!!!!
!! TFSI Koddermann
Nt   0.0   -0.051   1.824   0.0   -0.0255   1.824
St   0.0   -0.075   2.291   0.0   -0.0375   2.291
Ot   0.0   -0.063   1.944   0.0   -0.0315   1.944
Ct   0.0   -0.020   1.768   0.0   -0.0100   1.768
Ft   0.0   -0.016   1.490   0.0   -0.0080   1.490
!! LI cation OPLS
Li   0.0   -0.0005  1.6107  0.0   -0.00025  1.6107
!! TIP3P
OW   0.0   -0.1521  1.7683  0.0   -0.07605  1.7683
HW   0.0   -0.0406  0.2245  0.0   -0.0203   0.2245
NBFIX
OW HW -0.0836 1.9927 -0.0075 1.9927

END
```

A sample CP2K configuration file. The basis set and potential files were used from the CP2K distribution.

```
&GLOBAL
  PROJECT WIS
  RUN_TYPE MD
  PRINT_LEVEL LOW
&END GLOBAL


&FORCE_EVAL
  METHOD Quickstep
  &DFT
    charge 0
    &MGRID
      CUTOFF 260
      NGRIDS 4
      REL_CUTOFF 40
    &END MGRID
    &QS
      EPS_DEFAULT 1.0E-12
      EPS_GVG 1.0E-6
      EPS_PGF_ORB 1.0E-6
    &END QS
    &SCF
      EPS_SCF 1.0E-6
      MAX_SCF 1500
      SCF_GUESS atomic
      &OT
      &END OT
    &END SCF
    &XC
      &XC_FUNCTIONAL PBE
      &END XC_FUNCTIONAL
      &VDW_POTENTIAL
        POTENTIAL_TYPE PAIR_POTENTIAL
        &PAIR_POTENTIAL
          TYPE DFTD3
          REFERENCE_FUNCTIONAL PBE
          PARAMETER_FILE_NAME dftd3.dat
          CALCULATE_C9_TERM T
        &END PAIR_POTENTIAL
      &END VDW_POTENTIAL
    &END XC
    &PRINT
      &MOMENTS SILENT
      &END MOMENTS
    &END PRINT
  &END DFT
  &SUBSYS
    &CELL
      ABC 19.6152 19.6152 19.6152
    &END CELL
    &TOPOLOGY
       &CENTER_COORDINATES T
       &END CENTER_COORDINATES
       COORD_FILE_FORMAT XYZ
       COORD_FILE_NAME geo.xyz
    &END TOPOLOGY
```

```
    &KIND S
      BASIS_SET DZVP-MOLOPT-GTH-q6
      POTENTIAL GTH-PBE-q6
    &END KIND

    &KIND O
      BASIS_SET DZVP-MOLOPT-GTH-q6
      POTENTIAL GTH-PBE-q6
    &END KIND

    &KIND C
      BASIS_SET DZVP-MOLOPT-GTH-q4
      POTENTIAL GTH-PBE-q4
    &END KIND

    &KIND N
      BASIS_SET DZVP-MOLOPT-GTH-q5
      POTENTIAL GTH-PBE-q5
    &END KIND

    &KIND H
      BASIS_SET DZVP-MOLOPT-GTH-q1
      POTENTIAL GTH-PBE-q1
    &END KIND

    &KIND F
      BASIS_SET DZVP-MOLOPT-GTH-q7
      POTENTIAL GTH-PBE-q7
    &END KIND

    &KIND Li
      BASIS_SET DZVP-MOLOPT-SR-GTH-q3
      POTENTIAL GTH-PBE-q3
    &END KIND


 &END SUBSYS
&END FORCE_EVAL


&MOTION
  &MD
    ENSEMBLE NVT
    STEPS 100000
    TIMESTEP 1.0
    TEMPERATURE 298
    &THERMOSTAT
      &NOSE
        LENGTH 3
        YOSHIDA 3
        TIMECON 100.
        MTS 2
      &END NOSE
    &END
  &END MD
&END MOTION
```
